# “What can go wrong during thoracic endovascular aortic repair for type B aortic dissection”

**DOI:** 10.1016/j.jvscit.2024.101657

**Published:** 2024-10-23

**Authors:** Zachary Rengel, Gregory Magee

**Affiliations:** Division of Vascular Surgery and Endovascular Therapy, Keck Medical Center of the University of Southern California, Los Angeles, CA

Thoracic endovascular aortic repair (TEVAR) has become the gold standard treatment for patients with type B aortic dissections (TBAD) requiring surgical intervention.^1^, ^2^, ^3^ However, TEVAR for TBAD (TEVAR-AD) has several nuances that are distinct from treatment of other thoracic aortic pathology such as aneurysms and blunt thoracic aortic injuries. The aim of this article is to summarize the factors that can go awry during TEVAR-AD and how to recognize and correct these complications.

## Unintentional false lumen deployment

**Fig 1:**
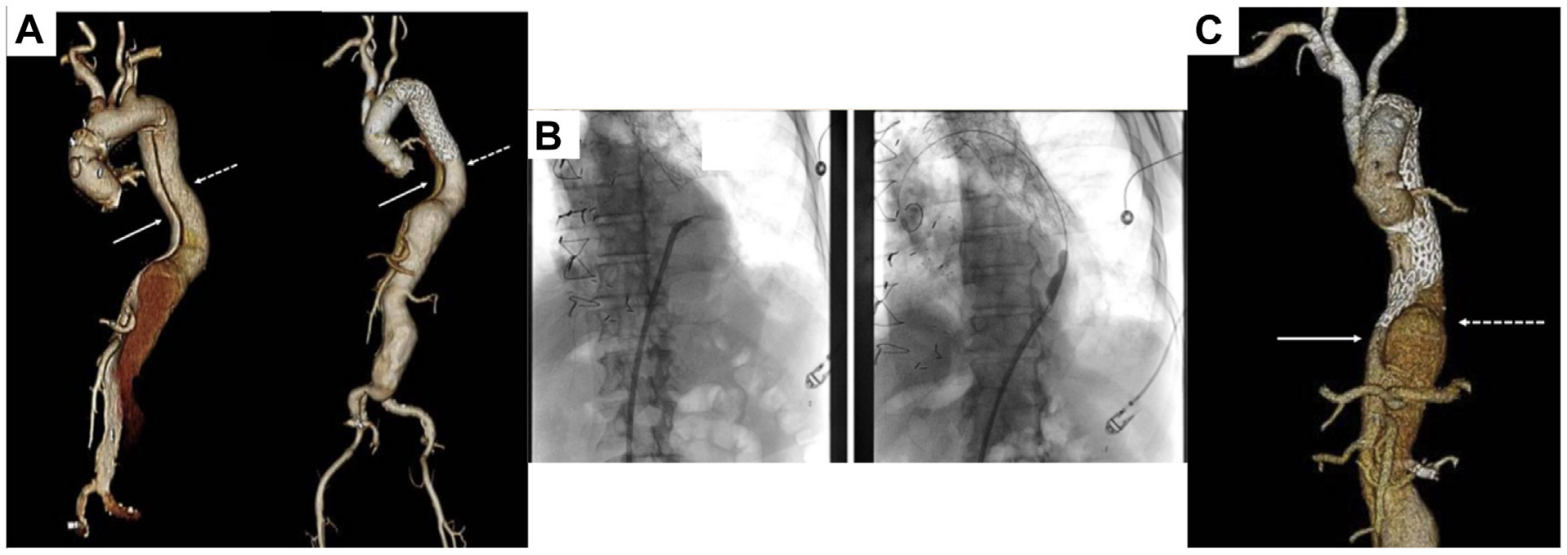
**(A)** Reconstructed three-dimensional computed tomography angiography (CTA) scan showing the pre- and postoperative images of patient with residual descending thoracic aortic dissection with subsequent false lumen deployment of frozen elephant trunk. The *solid arrows* point to the true lumen, whereas the *dashed arrows* point to the false lumen. **(B)** PowerWire (Baylis Medical) piercing through septum, cannulation of prior thoracic endovascular aortic repair endograft (TEVAR), and ballooning of septal fenestration. **(C)** Postoperative reconstructed three-dimensional CTA showing appropriate apposition of thoracic endovascular aortic repair (TEVAR) extending into the true lumen. (Reproduced from Plotkin A, Hanks SE, Han SM, Fleischman F, Weaver FA, Magee GA. Endovascular septal fenestration using a radiofrequency wire to salvage inadvertent false lumen deployment of a frozen elephant trunk stent graft. J Vasc Surg Cases Innov Tech 2019;5:553-556).

Inadvertent false lumen deployment of the endograft can be a devastating complication and is likely under-reported.^4^^,^^5^ This complication occurs because, in addition to the proximal entry tear, there are often other distal septal fenestrations between the true and false lumen that can occur when one or more of the visceral, renal, or intercostal arteries come off the false lumen. When the wire is being advanced from the femoral arteries into the ascending aorta, the wire may pass through these fenestrations. This can occur without the knowledge of the operator because it may not always be possible to recognize this on fluoroscopy. If the wire traverses from the true lumen in the arch to the false lumen distally, the endograft can be deployed into the false lumen which may result in visceral, renal, and/or lower extremity malperfusion (Fig 1, *A*).^6^ This complication can be prevented by always using intravascular ultrasound (IVUS) to confirm that the wire is in the true lumen throughout the entire course of the aorta. IVUS may also be helpful at confirming the location of branches and fenestrations and to help size the aorta during systole when it is at its greatest diameter.

When false lumen deployment has occurred, it is vital that it is recognized promptly. Close comparison of pre- and post-endograft deployment is fundamental to ensure that all visceral, renal, and iliac arteries are perfused, and that the proximal false lumen is not filling. When false lumen stenting occurs, there are several methods to enable bridging the stent back into the true lumen.

True lumen access should be obtained, and a fenestration can be created distal to the false lumen stent (Fig 1, *B*). Several techniques have been described to create this fenestration of the dissection septum from true to false lumen including using the stiff back end of a wire, radiofrequency wire, and in situ laser fenestration.^4^^,^^7^^,^^8^ IVUS should also be performed to confirm the wire location in the true lumen. This wire is then exchanged for a stiff wire and the fenestration can then be ballooned to enable placement of another thoracic endograft to bridge from the false lumen stent across the septum and into the true lumen (Fig 1, *C*).

## Retrograde type A dissection

**Fig 2:**
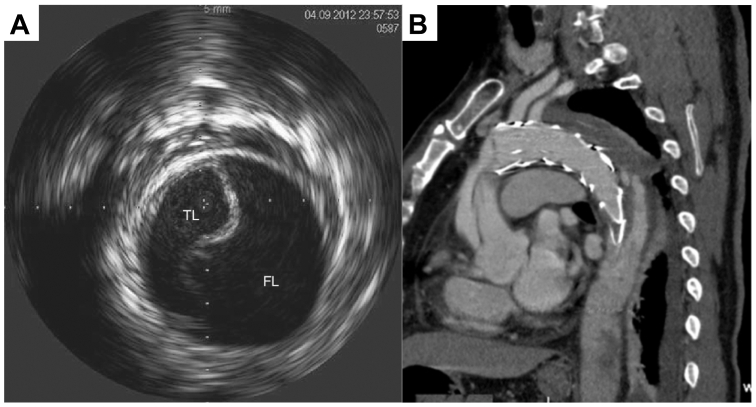
**(A)** Example of intravascular ultrasound (IVUS) confirmation of retrograde type A dissection (RTAD) in aortic arch. **(B)** CTA of retrograde type A dissection after thoracic endovascular aortic repair (TEVAR) repair. (Reproduced from Wendt D, Thielmann M, Melzer A, et al. The past, present and future of minimally invasive therapy in endovascular interventions: a review and speculative outlook. Minim Invasive Ther Allied Technol 2013;22:242-253. Ma T, Dong ZH, Fu WG, et al. Incidence and risk factors for retrograde type A dissection and stent graft-induced new entry after thoracic endovascular aortic repair. J Vasc Surg 2018;67:1026-1033.e2.).

One of the most feared complications of TEVAR-AD is a retrograde type A dissection (RTAD). Most retrograde dissections happen in the first few days to weeks postoperatively, but we have seen this occur acutely at the time of TEVAR-AD (Fig 2, *A* and *B*). Completion angiography was concerning, and retrograde dissection was confirmed with IVUS and transesophageal echocardiography. If this is noticed acutely, the patient should be taken emergently for open repair by cardiothoracic surgery. In our case, the patient remained hemodynamically stable, and the patient underwent successful repair. Several factors have been hypothesized to cause RTAD including oversizing of the proximal thoracic endograft, bare metal proximal struts, and landing the proximal end of the graft in disease aorta.^9^^,^^10^ One recommendation to prevent RTAD is to oversize the stent by 0% to 10% as opposed to 10% to 20% for thoracic aneurysm TEVAR cases. In our case, it was caused by a proximal extender endograft that propagated anteriorly during deployment, confirmed at the time of open repair with one of the struts at the entry tear. If cardiothoracic surgery is not immediately available, then the patient should be transferred rapidly to a center that can perform emergency open repair.

## Stent graft-induced new-entry

**Fig 3:**
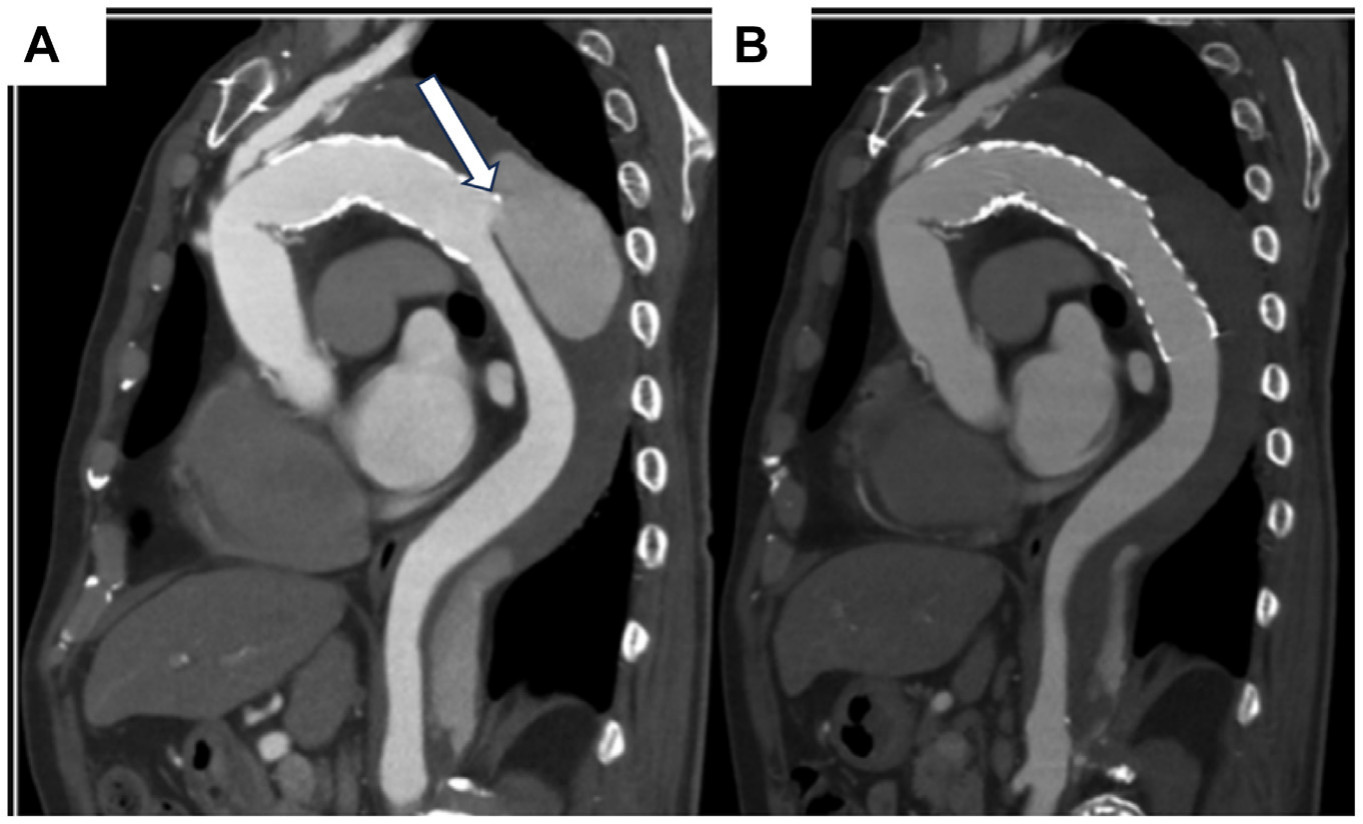
**(A)** Arrow pointing at distal stent graft-induced new entry (SINE) after thoracic endovascular aortic repair (TEVAR) **(B)** Follow-up computed tomography angiography (CTA) after TEVAR distal extension covering the SINE. (Reproduced from Nomura Y, Tonoki S, Kawashima M, et al. Distal stent graft-induced new entry after total arch replacement with frozen elephant trunk for aortic dissection. Ann Vasc Dis 2021;14:362-367.).

Stent graft-induced new entry (SINE) can occur when the distal aspect of the thoracic endograft exerts sufficient pressure on the dissection septum that it causes a new tear at that location (Fig 3, *A*). This prevents the goal of TEVAR, which is sealing the entry tear to promote false lumen thrombosis, and in the acute phase, a SINE can result in complete distal malperfusion if the septum tears and obstructs the aorta distal to the endograft. SINE is more likely to occur if treating acute TBADs early when the septum is most fragile. We recommend waiting 2 weeks or at least 3 days after the dissection occurs to perform TEVAR-AD unless the patient has rupture or malperfusion symptoms. Furthermore, we recommend using tapered endografts or placing a smaller distal endograft and “building up” to create a functional tapered graft, especially when the true lumen is small. Using a bare metal dissection stent distal to the thoracic endograft does not prevent SINE, but it does prevent the devastating complication of complete aortic or visceral obstruction from a SINE. If SINE is found during surveillance imaging and the patient is asymptomatic, they can be managed medically; however, if it is associated with significant aortic growth defined as diameter expansion of 5 mm or more during a 6-month period, repair can be performed with a distal extension of the TEVAR (Fig 3, *B*).^10^

## Aortic rupture

**Fig 4:**
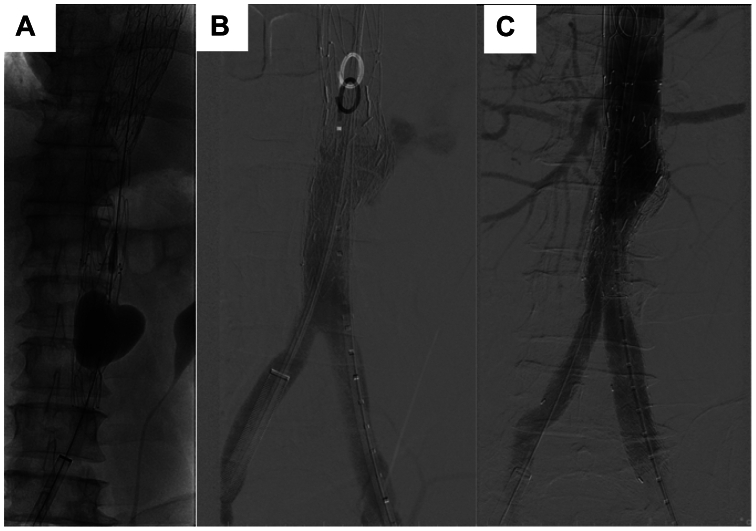
**(A)** Ballooning of infrarenal aorta. **(B)** Rupture of infrarenal aorta observed on post-balloon angiography. **(C)** Post-endovascular aortic repair (EVAR) angiogram with adequate seal zones and renal artery flow.

Another devastating complication of TEVAR-AD is aortic rupture. The STABILISE technique has been advocated as a method to enable relamination of the distal aortic dissection.^11^ One major concern about this technique is that the force needed by the aortic molding/occlusion balloon to tear the dissection septum can cause an aortic rupture. This has been reported, and we have seen this occur when using a balloon that is oversized to the overall diameter of the aorta. If the balloon expands beyond the area of the bare metal stents, rupture should be presumed (Fig 4, *A* and *B*). Immediately after identifying a rupture, endovascular repair should be performed with a TEVAR distal extension or EVAR depending on the location of the rupture (Fig 4, *C*).^12^ If operators choose to perform the STABILISE technique, we strongly advise using a semi-compliant balloon sized to the smallest total aortic diameter to prevent rupture.

### Coverage/shuttering of left common carotid or left subclavian arteries

**Fig 5:**
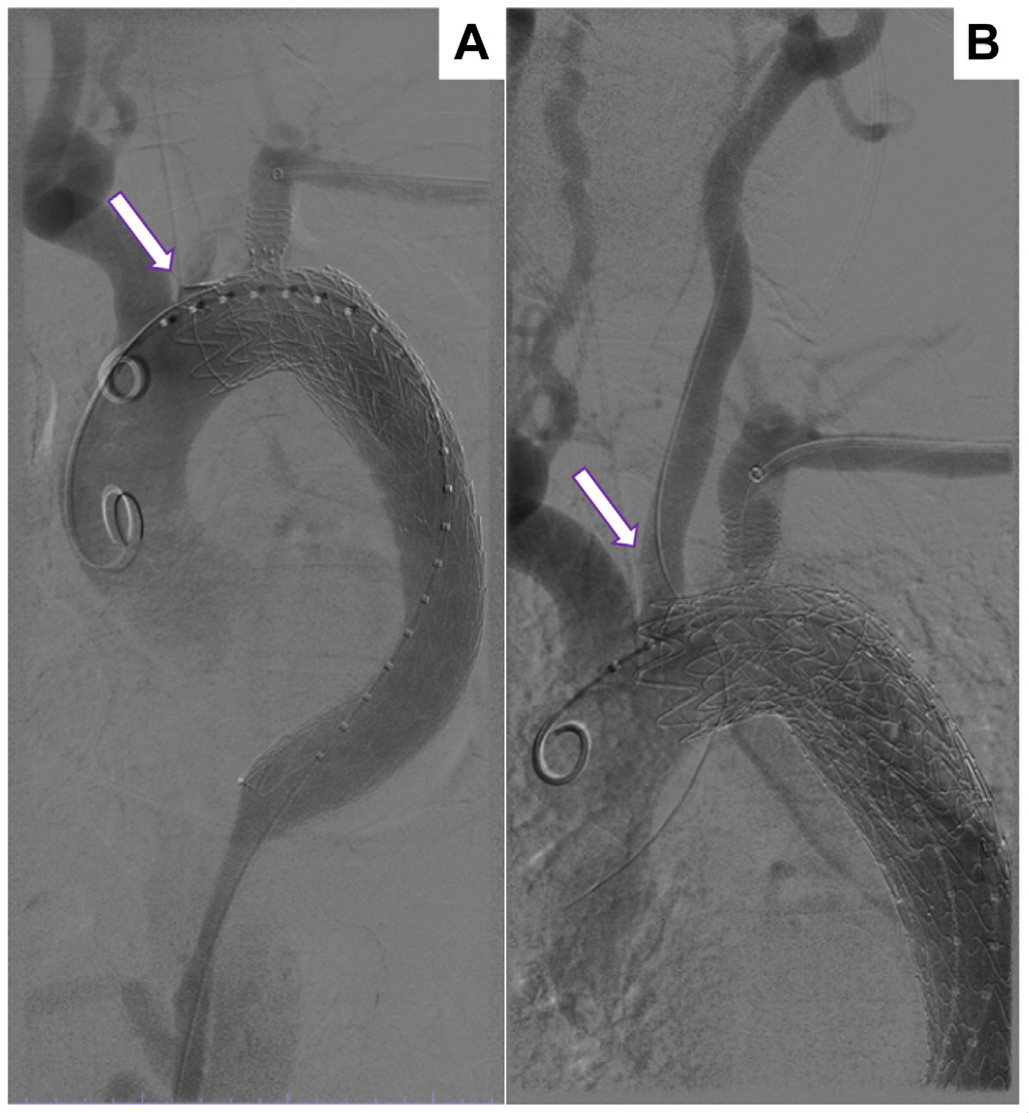
**(A)** Thoracic endovascular aortic repair (TEVAR) deployment in Zone 2 with shuttering of left common carotid artery (*arrow*) **(B)** Angiogram after ballooning of the origin left common carotid artery. Brisk flow through artery was confirmed (*arrow*).

The most common location of the proximal entry tear in TBAD is at or just distal to the left subclavian artery. The primary aim of TEVAR-AD is to cover the proximal entry tear and thus promote false lumen thrombosis. Therefore, covering the left subclavian artery for a zone 2 repair is frequently required.^13^ Due to the risk of stroke, spinal cord ischemia and left upper extremity ischemia with left subclavian artery (LSCA) coverage, the Society for Vascular Surgery (SVS) recommends routine preoperative revascularization (GRADE 2, level C).^14^ The Thoracic Branched Endograft (WL Gore) has been approved as an endovascular incorporation of the LSCA during zone 2 TEVAR.^13^ Of note, in acute thoracic emergencies, including acute aortic dissections, the SVS guidelines recommend that LSCA revascularization should be individualized according to patient anatomy, urgency of the procedure, and surgical expertise to LSCA revascularization.^14^ For aortic dissections that require Zone 2 coverage, a TEVAR that is deployed too proximally into Zone 2 can cover or shutter the origin of the left common carotid artery, which can lead to a stroke if not identified and corrected.^15^ We saw this complication occur during our early use of the Conformable CTAG device (WL Gore), as the inner sleeve, which is 50% of the final graft diameter, can obstruct branches on the outer curve that are only covered by the bare metal proximal struts of the graft (Fig 5, *A*). Correcting this complication can be performed by either cutting down on the left common carotid and performing a retrograde snorkel stent or subclavian to carotid bypass, or by accessing the left common carotid antegrade and placing a stent or ballooning at its origin. Both IVUS and angiography can be used to confirm that the origin of the left common carotid is no longer shuttered (Fig 5, *B*). We strongly recommend against trying to use an aortic molding or occlusion balloon to pull back the thoracic endograft as this can lead to even worse complications such as aortic rupture or retrograde dissection.

## Conclusion

A detailed understanding of preoperative imaging and anatomy, TEVAR deployment behavior, and rescue techniques are imperative to both prevent and remedy potential complications that arise during or following TEVAR-AD.

## Disclosures

G.A.M. is a consultant for W. L. Gore & Associates and Silk Road Medical.
